# The Polluters: The Making of Our Chemically Altered Environment

**DOI:** 10.1289/ehp.119-a404

**Published:** 2011-09-01

**Authors:** Gregory J. Howard

**Affiliations:** Gregory J. Howard in an environmental health scientist and assistant professor of environmental studies at Dickinson College. His research focuses on methods for assessing and modeling interactions of multiple toxic exposures as well as on monitoring of community-identified environmental pollutants. He is co-author of an upcoming *Health Studies Guide* for communities concerned about environmentally related diseases.

Histories of environmental pollution usually begin with the Donora smog, which in 1948 first opened the nation’s eyes to the consequences of pollution, or emphasize the dramatic progress made since the watershed publication of *Silent Spring* in 1962. Ross and Amter’s ambitious new book, *The Polluters*, attempts to tell a deeper history, one dating back to the beginnings of the Industrial Revolution. Most important, the authors trace in well-documented detail the development of tactics used by early polluters—tactics later adopted and codified by the modern chemical industry.

Corporate malfeasance is an easy target, but, despite its title, much of the book is concerned not with the polluters but with their enablers. The most interesting among these are the highly intelligent, often well-meaning scientists and engineers who simply thought they knew best how to manage pollution and who, like their employers, wanted to do their jobs without interference. Many intriguing characters emerge in these pages; unfortunately, in their sprint through over a century of history, Ross and Amter are rarely able to flesh them out.

Perhaps the most influential of the enabler-scientists was Robert Kehoe. As a newly minted physician, Kehoe was tasked with investigating deaths in General Motors’ pilot tetraethyl lead plant, the “House of Butterflies,” named for the hallucinations common to its workers; his success there led him in 1930 to found the Kettering Laboratory, named after GM’s own director of research. With nearly unlimited funds from his industrial supporters—and with the promise of corporate review of publications—Kehoe, arguing from patently flawed studies that lead is a normal part of the human environment, pioneered the argument that synthetic chemicals such as tetraethyl lead should be banned only if an “actual hazard” could be shown; conveniently, he was one of the few with the means to do so. This requirement has driven pollution regulation ever since.

An entirely different approach had been offered a generation before by the eminent 19th-century physicist Lord Kelvin, who headed a Royal Commission investigating a 1900 outbreak of arsenic poisoning among Manchester beer drinkers. With few data on low-dose effects of arsenic, Kelvin took an approach we would today label “precautionary”: “In the absence of fuller knowledge than is at present available, we are not prepared to allow that it would be right to declare any quantity of arsenic, however small, as admissible in beer or in any food.” The British government instituted Kelvin’s strict limits, which soon became standard throughout Europe, but American growers argued that meeting this “world tolerance” would bankrupt them. This put the American government, hobbled by the need to demonstrate an “actual hazard” before acting, in an awkward position. The Agriculture Department quietly began testing fruit destined for export, but not for domestic consumption; at the same time, the nascent Food, Drug, and Insecticide Administration set less protective domestic standards, without publicly disclosing them.

The conflict of views between Kehoe and Kelvin—between proof and precaution—is at the core at the history of pollution. The twin arguments of “actual hazard” and economic necessity developed, over the 20th century, into the highly effective tactic Ross and Amter call “spill, study, and stall.” Economic arguments often trumped all other concerns, and the willingness of intelligent public health scientists to concede these battles has been distressing. Johns Hopkins’ Abel Wolman, pioneer of drinking-water chlorination, argued before the Senate that it was “not fair” to require controls on pollution if the “investment return did not compare favorably with the return on other capital projects.” To the modern scientist, aware of the strides made in recent decades under federal regulation, Wolman’s view represents a capitulation to a false economics and a failure of the imagination. The same point of view hindered DuPont’s post-Donora attempt to address its own waste management issues, in an episode Ross and Amter relate in compelling detail. Predictably, the effort floundered because, in the absence of any corporate-wide metric for environmental progress, division managers refused to install waste-treatment equipment “not fully justified by savings.” Ironically, the federal regulations that DuPont so bitterly opposed would have solved the problem that corporate headquarters was unable to address internally; but industrial leaders failed, or refused, to see that regulation could help create a level playing field for their activities.

The attempts by the states to regulate pollution proceeded with excruciating slowness until Donora, and federal regulation gained traction only after *Silent Spring*. From this point, however, *The Polluters* races toward its conclusion with sometimes frustrating speed. A brief clause inserted into a bill by New York State Representative James Delaney, and its consequences for food safety, merits a few sentences; the chemical industry’s attacks on Rachel Carson less than a paragraph. The uneven pacing is sometimes disorienting: Indeed, in some cases the authors appear to have let their extensive research dictate the narrative, as in an odd digression describing the purchase prices of homes of Bureau of Mines chief Royd Sayers. The brevity of individual chapters makes the detailed and carefully documented case histories—lavishly supported by colorful quotations from primary documents—accessible reading. For the most part, however, Ross and Amter prefer to steer clear of drawing lessons from the stories they tell. Readers may find themselves wishing for more analysis than the paragraph or two that closes each chapter.

Other works have told parts of this story in more depth, and with more attention to the personalities involved. *The Polluters* lacks the coherence of more focused histories such as Devra Davis’s *The Secret War on Cancer* or Rosner and Markowitz’s masterly *Deceit and Denial*. But Ross and Amter’s contribution is to weave together into a single, readable narrative the long and sordid history of the struggle over environmental regulation, and readers attuned to the environmental debates of the 21st century will be struck by the familiarity of the tactics developed by the polluters who came before.

